# Conductance of Graphene Nanoribbon Junctions and the Tight Binding Model

**DOI:** 10.1007/s11671-010-9791-y

**Published:** 2010-10-07

**Authors:** Y Wu, PA Childs

**Affiliations:** 1 School of Electronic, Electrical and Computer Engineering, University of Birmingham, B15 2TT, Birmingham, UK

**Keywords:** Graphene nanoribbon junction, Tight binding, Conductance, Band structure

## Abstract

Planar carbon-based electronic devices, including metal/semiconductor junctions, transistors and interconnects, can now be formed from patterned sheets of graphene. Most simulations of charge transport within graphene-based electronic devices assume an energy band structure based on a nearest-neighbour tight binding analysis. In this paper, the energy band structure and conductance of graphene nanoribbons and metal/semiconductor junctions are obtained using a third nearest-neighbour tight binding analysis in conjunction with an efficient nonequilibrium Green's function formalism. We find significant differences in both the energy band structure and conductance obtained with the two approximations.

## Introduction

Since the report of the preparation of graphene by Novoselov et al. [1] in 2004, there has been an enormous and rapid growth in interest in the material. Of all the allotropes of carbon, graphene is of particular interest to the semiconductor industry as it is compatible with planar technology. Although graphene is metallic, it can be tailored to form semiconducting nanoribbons, junctions and circuits by lithographic techniques. Simulations of charge transport within devices based on this new technology exploit established techniques for low dimensional structures [2,3]. The current flowing through a semiconducting nanoribbon formed between two metallic contacts has been established using a nonequilibrium Green's Function (NEGF) formalism based coupled with an energy band structure derived using a tight binding Hamiltonian [4-7]. To minimise computation time, the nearest-neighbour tight binding approximation is commonly used to determine the energy states and overlap is ignored. This assumption has also been used for calculating the energy states of other carbon-based materials such as carbon nanotubes [8] and carbon nanocones [9]. Recently, Reich et al. [10] have demonstrated that this approximation is only valid close to the *K* points, and a tight binding approach including up to third nearest-neighbours gives a better approximation to the energy dispersion over the entire Brillouin zone.

In this paper, we simulate charge transport in a graphene nanoribbon and a nanoribbon junction using a NEGF based on a third nearest-neighbour tight binding energy dispersion. For transport studies in nanoribbons and junctions, the formulation of the problem differs from that required for bulk graphene. Third nearest-neighbour interactions introduce additional exchange and overlap integrals significantly modifying the Green's function. Calculation of device characteristics is facilitated by the inclusion of a Sancho-Rubio [11] iterative scheme, modified by the inclusion of third nearest-neighbour interactions, for the calculation of the self-energies. We find that the conductance is significantly altered compared with that obtained based on the nearest-neighbour tight binding dispersion even in an isolated nanoribbon. Hong et al. [12] observed that the conductance is modified (increased as well as decreased) by the presence of defects within the lattice. Our results show that details of the band structure can significantly modify the observed conductivities when defects are included in the structure.

## Theory

**Figure 1:**
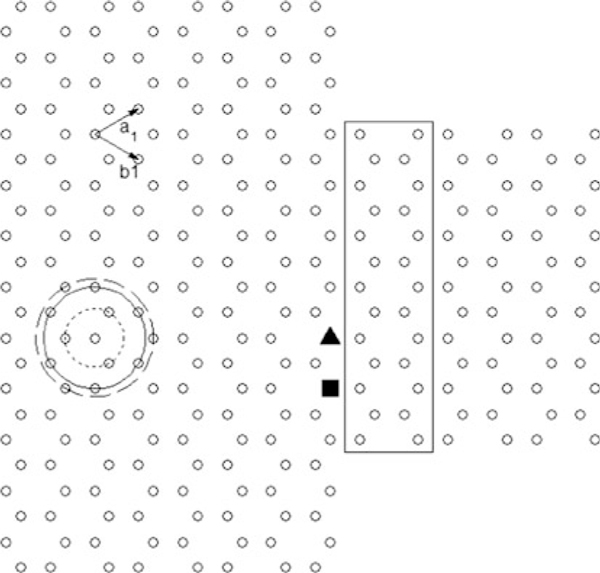
**Armchair-edge graphene metal (index *N* = 23)/semiconductor (index *N* = 13) junction**. The *rectangle* shows the semiconductor unit cell, and the *concentric circles* of increasing radius show first, second and third nearest-neighbours, respectively.

The basis for our analysis is the hexagonal graphene lattice shown in Figure 1. **a**_**1**_ and **b**_**1**_ are the principal vectors of the unit cell containing two carbon atoms belonging to the two sub-lattices. Atoms on the concentric circles of increasing radius correspond to the nearest-neighbours, second nearest-neighbours and third nearest-neighbours, respectively.

Saito et al. [8] derived the dispersion relation below using a nearest-neighbour tight binding analysis including the overlap integral *s*_0_.

(1) $$E^{\pm }(\text{k})=\frac{\varepsilon_{2p}\mp \gamma_{0}\sqrt{f(\text{k})}}{1\mp s_{0}\sqrt{f(\text{k})}}$$

Here, *f*(*k*) = 3 + 2 cos **k** · **a_1_** +2 cos **k** · **b_1_** + 2 cos **k** · (**a_1_** - **b_1_**) and the parameters, *ε*_2*p*_, *γ*_0_ and *s*_0_ are obtained by fitting to experimental results or ab initio calculations.

Most analyses of charge transport in graphene-based structures simplify the result further by ignoring *s*_0_. Reich et al. [10] derived the dispersion relation for graphene based on third nearest-neighbours. In this work, the energy band structure of a graphene nanoribbon including third nearest-neighbour interactions is obtained from the block Hamiltonian and overlap matrices given below for the unit cell defined by the rectangle in Figure 1.

(2) $$\begin{array}{l} E\left[\begin{array}{ccccc} S_{0,0} & S_{0,1} & & & \\ & \ddots & & & \\ & S_{n,n-1} & S_{n,n} & S_{n,n+1} & \\ & & & \ddots & \\ & & & S_{N,N-1} & S_{N,N} \end{array}\right]\left[\begin{array}{c} \phi_{0}\\ \vdots \\ \phi_{n}\\ \vdots \\ \phi_{N} \end{array}\right]\\ =\left[\begin{array}{ccccc} H_{0,0} & H_{0,1} & & & \\ & \ddots & & & \\ & H_{n,n-1} & H_{n,n} & H_{n,n+1} & \\ & & & \ddots & \\ & & & H_{N,N-1} & H_{N,N} \end{array}\right]\left[\begin{array}{c} \phi_{0}\\ \vdots \\ \phi_{n}\\ \vdots \\ \phi_{N} \end{array}\right] \end{array}$$

For the *n*th row of the above equation, we have

(3) $$\begin{array}{l} H_{n,n-1}\phi_{n-1}+H_{n,n}\phi_{n}+H_{n,n+1}\phi_{n+1}\\ \;-E(S_{n,n-1}\phi_{n-1}+S_{n,n}\phi_{n}+S_{n,n+1}\phi_{n+1})=0 \end{array}$$

Considering the energy dispersion in the direction of charge transport, the Bloch form of the wavefunction ensures that *φ*_*n*_~e^i*kn*^. Substitution of *φ*_*n*_ into the above equation yields the secular equation

(4) $$\begin{array}{l} \det[H_{n,n-1}{\text{e}}^{-\text{i}k}+H_{n,n}+H_{n,n+1}{\text{e}}^{\text{i}k}\\ \;-E(S_{n,n-1}{\text{e}}^{-\text{i}k}+S_{n,n}+S_{n,n+1}{\text{e}}^{\text{i}k})]=0 \end{array}$$

In the case of first nearest approximation without orbital overlap, *S*_*n*,*n*-1_ and *S*_*n*,*n*+1_ are empty matrices. To facilitate comparison with published results, we use an armchair-edge with index [13]*N* = 13 as our model nanoribbon. In the paper by Reich, tight binding parameters were obtained by fitting the band structure to that obtained by ab initio calculations. Recently, Kundo [14] has reported a set of tight biding parameters based on fitting to a first principle calculation but more directly related to the physical quantities of interest. These parameters have been utilised in our calculation and are presented below for third nearest-neighbour interactions (Table 1).

**Table 1:** Tight binding parameters [14]

Neighbours	***E***_**2**_***p*(eV)**	***γ***_**0**_**(eV)**	***γ***_**1**_**(eV)**	***γ***_**2**_**(eV)**	***s***_**0**_	***s***_**1**_	***s***_**2**_
3rd-nearest	-0.45	-2.78	-0.15	-0.095	0.117	0.004	0.002

**Figure 2:**
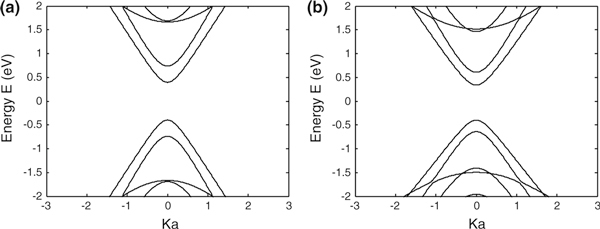
**Energy band structure of an *N* = 13 armchair graphene nanoribbon, *(a)* obtained from the first nearest-neighbour tight binding method and *(b)* including third nearest-neighbours**.

Figure 2 compares the energy band structure of the modelled armchair-edge graphene nanoribbon obtained from the first nearest-neighbour tight binding method with that obtained by including up to third nearest-neighbours. Agreement is reasonable close to the *K* point but significant discrepancies occur at higher energies.

## Conductance of Graphene Nanoribbons and Junctions

Conductance in graphene nanoribbons and metal/semiconductor junctions is determined using an efficient nonequilibrium Green's function formalism described by Li and Lu [15]. The retarded Green's function is given by

(5) $$G={\left[E^{+}S-H-\Sigma^{L}-\Sigma^{R}\right]}^{-1}$$

**Figure 3:**
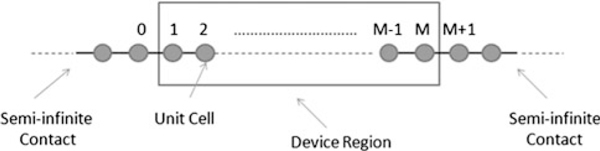
**Schematic showing the unit cell labelling used to formulate the Green's function**.

Here, *E*^+^ = *E* + i*η* and *η* is a small positive energy value (10^-5^ eV in this simulation) which circumvents the singular point of the matrix inversion [16]. *H* is a tight binding Hamiltonian matrix including up to third nearest-neighbours, and *S* is the overlap matrix. Open boundary conditions are included through the left and right self-energy matrix elements, *σ*^*L*.*R*^. The self-energies are independently evaluated through an iterative scheme described by Sancho et al. [11], modified to include third nearest-neighbour interactions. Determination of the retarded Green's function through equation 5 is facilitated by the inclusion of the body of the device in the right-hand contact through the recursive scheme described in ref. [15]. We will now outline the numerical procedure for deriving the conductance with third nearest-neighbour interactions included. Figure 3 shows a schematic of the unit cell labelling used to formulate the Green's function.

We calculate the surface retarded Green's functions of the left and right leads by

(6) $$g_{0,0}^{L}={\left[E^{+}S_{0,0}-H_{0,0}-(E^{+}S_{0,-1}-H_{0,-1})\tilde{\theta }\right]}^{-1}$$

(7) $$g_{M+1,M+1}^{R}={\left[E^{+}S_{0,0}-H_{0,0}-(E^{+}S_{-1,0}-H_{-1,0})\theta \right]}^{-1}$$

where *θ* and $\tilde{\theta }$ are the appropriate transfer matrices calculated from the following iterative procedure.

(8) $$\theta =t_{0}+\tilde{t_{0}}t_{1}+\tilde{t_{0}}\tilde{t_{1}}t_{2}+\cdots +\tilde{t_{0}}\tilde{t_{1}}\tilde{t_{2}}\cdots t_{n}$$

(9) $$\tilde{\theta }=\tilde{t_{0}}+t_{0}\tilde{t_{1}}+t_{0}t_{1}\tilde{t_{2}}+\cdots +t_{0}t_{1}t_{2}\cdots \tilde{t_{n}}$$

where *t*_*i*_ and $\tilde{t_{i}}$ are defined by

(10) $$t_{i}={\left(I-t_{i-1}\tilde{t_{i-1}}-\tilde{t_{i-1}}t_{i-1}\right)}^{-1}t_{i-1}^{2}$$

(11) $$\tilde{t_{i}}={\left(I-t_{i-1}\tilde{t_{i-1}}-\tilde{t_{i-1}}t_{i-1}\right)}^{-1}{\tilde{t_{i-1}}}^{2}$$

and

(12) $$t_{0}={\left(E^{+}S_{0,0}-H_{0,0}\right)}^{-1}(E^{+}S_{0,-1}-H_{0,-1})$$

(13) $$\tilde{t_{0}}={\left(E^{+}S_{0,0}-H_{0,0}\right)}^{-1}(E^{+}S_{-1,0}-H_{-1,0})$$

The process is repeated until $t_{0}\tilde{t_{0}}<\delta$ with *δ* arbitrarily small. The nonzero elements of the self-energies $\Sigma_{1,1}^{L}$ and $\Sigma_{M,M}^{R}$ can be then obtained by

(14) $$\Sigma_{1,1}^{L}=(E^{+}S_{1,0}-H_{1,0})g_{0,0}^{R}(E^{+}S_{0,1}-H_{0,1})$$

(15) $$\Sigma_{M,M}^{R}=(E^{+}S_{0,1}-H_{0,1})g_{M+1,M+1}^{R}(E^{+}S_{1,0}-H_{1,0})$$

The conductance is obtained from the relation

(16) $$G(E)=\frac{2e^{2}}{h}T(E)$$

where the transmission coefficient is obtained from

(17) $$T\left(E\right)=Tr[\Sigma^{L}G\Sigma^{R}G^{†}],$$

with Γ^*L,R*^ = *i*[Σ^*L,R*^ - (Σ^*L,R*^)^†^.

**Figure 4:**
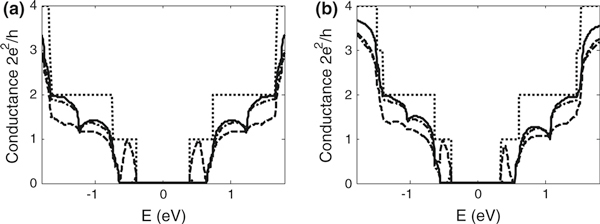
**Conductance vs Energy for the junction shown in Figure 1, a using first nearest-neighbour parameters and b using third nearest-neighbours parameters**. *Dotted lines* are for *N* = 13 armchair nanoribbon, *solid lines* are for ideal metal/semiconductor junctions, *dot*–*dash lines* and *dash lines* are for junctions with a single defect type A (*triangle* in Figure 1) and type B (*rectangle* in Figure 1) respectively.

Figure 4a, b compares the conductance of a graphene armchair-edge nanoribbon of index *N* = 13 and metal/semiconductor junction formed with the nanoribbon assuming first and third nearest-neighbour interactions, respectively. For graphene nanoribbons, differences are observed in the step-like structure, reflecting differences in the calculated band structure. When only first nearest-neighbour interactions are considered, the conductance of the conduction and valence bands is always symmetrical as determined by the formulation of the energy dispersion relation, equation 1. In the case of graphene nanoribbons, the conductance within a few electron volts of the Fermi energy is symmetrical for both first and third nearest-neighbour interactions. However, it is notable that at higher energies, overlap integrals introduced by third nearest-neighbour interactions result in asymmetry between the conductance in the conduction and valence bands. For metal/semiconductor junctions, significant differences in conductivity occur even at low energies due to mismatches of the sub-bands. Asymmetry in the conduction and valence band conductance (absent for first nearest-neighbour interactions) is also apparent when third nearest-neighbour interactions are included in the Green's function. Differences are also seen when defects are incorporated within a metal/semiconductor junction, an interesting system explored by Hong et.al. [12]. In this work, vacancies are introduced in the lattice at the positions marked by the solid rectangle and triangle in Figure 1 and the conductance obtained in each case. Hong et al. derive a coupling term associated with differences in band structure. For third nearest-neighbour, the solution to the coupling strength must be derived numerically.

## Conclusions

In this paper, we have determined the energy band structure of graphene nanoribbons and conductance of nanoribbons and graphene metal/semiconductor junctions using a NEGF formalism based on the tight binding method approximated to first nearest-neighbour and third nearest-neighbour. Significant differences are observed, suggesting the commonly used first nearest-neighbour approximation may not be sufficiently accurate in some circumstances. The most notable differences are observed when defects are introduced in the metal/semiconductor junctions.
